# High Interleukin-8 Levels Associated With Decreased Survival in Patients With Cirrhosis Following Transjugular Intrahepatic Portosystemic Shunt

**DOI:** 10.3389/fmed.2022.829245

**Published:** 2022-02-28

**Authors:** Guofeng Liu, Xiaoze Wang, Tingting Yang, Yuling Yan, Tong Xiang, Li Yang, Xuefeng Luo

**Affiliations:** ^1^Department of Gastroenterology and Hepatology, Sichuan University-University of Oxford Huaxi Joint Centre for Gastrointestinal Cancer, West China Hospital, Sichuan University, Chengdu, China; ^2^Department of Andrology/Sichuan Human Sperm Bank, West China Second University Hospital, Sichuan University, Chengdu, China

**Keywords:** cirrhosis, portal hypertension, systemic inflammation, interleukin-8 (IL-8), transjugular intrahepatic portal systemic shunt (TIPS)

## Abstract

**Background:**

Serum cytokines—reflecting systemic inflammation has been associated with the risk of decompensation and mortality in patients with cirrhosis. However, the role of systemic inflammation in patients with cirrhosis undergoing transjugular intrahepatic portosystemic shunt procedure remains unknown.

**Patients and Methods:**

Patients with cirrhosis who received transjugular intrahepatic portosystemic shunt between June 2015 and September 2017 were included. Portal and hepatic venous blood samples were obtained intraoperatively; serum cytokine levels (IL-10, IL-17A, IL-1RA, IL-8, and CXCL10) were measured in 105 patients. Associations with survival and other outcomes during long-term follow-up (median: 1,564 days) were assessed using logistic regression.

**Results:**

IL-17A and CXCL10 levels were higher in the portal than in the hepatic veins, whereas IL-1RA levels were higher in the hepatic than in the portal veins. However, IL-8 or IL-10 levels between hepatic and portal veins showed no differences. Multivariate analysis demonstrated that Child–Pugh scores (*P* = 0.017, HR: 1.484, 95% CI: 1.072–2.055) and IL-8 level in hepatic veins (*P* < 0.001, HR: 1.043, 95% CI: 1.019–1.068) were independent predictors for mortality during long-term follow-up, with an optimal cut-off of 5.87 pg/ml for IL-8 in hepatic veins. Patients with hepatic IL-8 levels < 5.87 pg/ml had significantly higher cumulative survival rates (98.4 vs. 72.9% at 1 year, 98.4 vs. 65.3% at 2 years, 96.7 vs. 60.3% at 3 years, 94.2 vs. 60.3% at 4 years; *P* < 0.0001).

**Conclusions:**

IL-8 levels in hepatic veins may reflect liver cirrhosis severity. Elevated IL-8 levels suggest shorter survival in patients receiving TIPS.

## Introduction

Portal hypertension, a major driver of complications in patients with cirrhosis, can cause hepatic inflammation, fibrosis, and angiogenesis, as well as aggravate the progression of cirrhosis (1). Previous studies have demonstrated that systemic inflammation (SI) increased with the severity of portal hypertension and circulatory dysfunction, and may be a key driver of clinical deterioration (2–4). SI and portal hypertension are linked by endothelial dysfunction and innate immune interactions within the sinusoidal niche of the injured liver (3). Recently, it was demonstrated that IL-6 and C-reactive protein levels correlated with the risk of first decompensation and reflected the degree of SI in patients with cirrhosis (5). Transjugular intrahepatic portosystemic shunt (TIPS) is a minimally invasive intervention used to effectively relieve portal hypertension and treat complications such as variceal bleeding and refractory ascites (6). However, it also increases the risk of liver failure, overt hepatic encephalopathy, and cardiac decompensation (7). The chemokine (C-X-C motif) receptor 3 (CXCR3) ligands (CXCL9, CXCL10, and CXCL11)—involved in SI—were identified as surrogate markers for the long-term survival of patients with cirrhosis receiving TIPS (8–10).

Interleukin-10 (IL-10), interleukin-17A (IL-17A), interleukin-1 receptor antagonist (IL-1RA), interleukin-8 (IL-8), and chemokine ligand 10 (CXCL10)—known markers of immune activity linked to specific parts of the immune response, particularly SI—were found to be variably elevated in patients with chronic liver disease (11–14). However, the role of other cytokines (IL-10, IL-17A, IL-1RA, IL-8) regarding the prognosis of patients with severe portal hypertension receiving TIPS remains poorly investigated. There remains a difference in serum cytokine levels between portal and hepatic veins; additionally, it is not clear whether cytokine levels in hepatic or portal veins can predict the clinical prognosis of patients with severe portal hypertension. A TIPS provides access to blood from the portal and hepatic veins; hence, our study aimed to evaluate and analyze the prognostic role of cytokine levels from the portal and hepatic veins in a cohort of patients with portal hypertension receiving TIPS.

## Materials and Methods

This retrospective study was approved by the ethical review board of West China Hospital; all patients provided written informed consent for the TIPS procedures. We enrolled 253 patients with cirrhosis and portal hypertensive complications who underwent the TIPS procedure between June 2015 and September 2017. Blood was successfully collected from the portal and hepatic veins of 127 patients. Patients who exhibited malignant tumors before (*n* = 16), or technical failure during the TIPS procedure (*n* = 6), were excluded; thus, 105 patients were included in the final analysis. Data were collected in a local database and retrospectively analyzed.

### TIPS Procedure

All TIPS procedures were performed by a single interventional radiologist with 10 years of experience. Generally, the right internal jugular vein was used to advance the TIPS set (RUPS-100; Cook Group, Bloomington, Indiana, United States) into the inferior vena cava, and then the right hepatic vein. Once the portal vein was successfully punctured and catheterized, direct portography was performed; the portosystemic pressure gradient (PPG) was then measured. The intrahepatic parenchymal tract was predilated with an 8 × 60 mm angioplasty balloon (Cordis, Hialeah, Florida, United States), followed by insertion of an 8-mm ePTFE-covered stent (Fluency Plus; BD, Franklin Lakes, New Jersey, United States). A second, bare stent (Cordis) or stent-graft (Fluency) was coaxially inserted if the first stent-graft could not maintain sufficient intrastent blood flow. Portography and PPG measurements were repeated after TIPS creation. Generally, visualized gastroesophageal varices were embolized using coils (Cook Group, Bloomington, Indiana, United States), and sometimes combined with glue (Guangzhou Baiyun, Guangdong, China).

### Assessment of Circulating Levels of Cytokines

Blood from the portal and the hepatic veins were collected as previously described (8). In brief, hepatic venous blood samples were taken before portal vein puncture, while portal venous blood samples were taken immediately after access into the portal vein. Blood samples were centrifuged at 3,000 rotations/min for 15 min at 4°C. Serums in the upper layer were stored at −80°C until they were required for cytokine analyses. Serum cytokines were assessed with the Luminex Magpix system with Milliplex Human Cytokine Magnetic Bead Panel (Merck Group, Darmstadt, Germany), according to the manufacturer's instructions. The cytokine levels were quantified in each undiluted serum sample in duplicate.

### Statistical Analysis

All statistical analyses in this study were performed by using SPSS version 23.0 (IBM, Chicago, IL, USA) and GraphPad Prism version 9.00 (GraphPad Software, La Jolla, USA). Data were presented as mean ± standard deviation (if normal distribution) or median or interquartile (if non-normal distribution), and classification variables were recorded as absolute values and percentages. The Student's *t-*test was used to compare continuous variables (normal distribution), but the non-normal distribution used Wilcoxon Signed Rank Test or the Kruskal-Wallis test, Variance analysis, and Fisher's test to compare categorical variables. Pearson correlation test (normal distribution) or Spearman correlation analysis (skewed distribution) is used to analyze the correlation between quantitative variables. Kaplan–Meier curves were used to analyse the survival rates of patients using the log-rank test. Multivariable analysis of independent predictors for survival was performed by a backward stepwise Cox proportional hazards regression model. Statistical analysis using the two-sided test, with *P* < 0.05 for the difference was significant.

## Results

### Patient Characteristics

The patients included in this study were predominantly male (70.5%), with a median age of 49 years (Table 1). The median Child–Pugh score was 7 points, while most patients exhibited class B cirrhosis; the median model for end stage liver disease (MELD) score was 10. Most patients (75.2%) had cirrhosis due to chronic virus hepatitis infection (*n* = 79); the remaining 24.8% patients had alcohol cirrhosis (*n* = 10), primary biliary cirrhosis (*n* = 8), autoimmune hepatitis (*n* = 4), overlap syndrome (*n* = 1), Budd-Chiari syndrome (*n* = 2), or schistosome (*n* = 1). Indications for the TIPS procedure were secondary prophylaxis for recurrent bleeding (*n* = 97), acute variceal bleeding (*n* = 7), and refractory ascites (*n* = 1).

**Table 1:** Baseline characteristic of the study population.

**Parameters**	***N* = 105**
Age (years)	49 (43–60)
Male/female	74/31
**Etiology**	
Viral	79
Alcohol	10
Autoimmune	13
Others	3
**Indication for TIPS**	
Controlling acute variceal bleeding	7
Prevention of variceal bleeding	97
Refractory ascites	1
PPG (mmHg)	22 (19–27)
TBil (umol/L)	21.0 (15.3–29.5)
Albumin (g/L)	32.7 (29.2–37.3)
ALT (U/L)	21 (16–36)
AST (U/L)	32 (25–48)
Platelet count ( × 10^9^/L)	62 (40–81.5)
INR	1.24 (1.13–1.39)
Serum creatinine (umol/L)	69 (59–76)
BUN	5.1 (3.8–6.7)
WBC ( × 10^9^/L)	2.95 (2.16–4.48)
**Ascites**	
No	27
Small	26
Medium	25
Large	27
**PVT**	
Yes	29
No	76
**HE before TIPS**	
Yes	0
No	105
Child-pugh scores	7 (6–8)
A	32
B	67
C	6
MELD scores	10 (9–11)

*Data are shown as median and ranges for continuous variables or total number for categorical variables. PPG, portosystemic pressure gradient; TBil, total bilirubin; AST, aspartate transaminase; ALT, alanine transaminase; INR, international normalized ratio; BUN, blood urea nitrogen; WBC, white blood cells; PVT, portal vein thrombosis; HE, hepatic encephalopathy; MELD, model for end stage liver disease; TIPS, transjugular intrahepatic portosystemic shunt*.

In our cohort, the median portal-systemic pressure gradient decreased from 22 (15–23) mmHg before, to 9 (7–11) mmHg after the TIPS procedure.

The median follow-up period was 1,564 days after the TIPS procedure. Of 105 patients, variceal rebleeding occurred in 30. Thirty-seven patients experienced hepatic encephalopathy, and 21 patients died. Nine patients died due to gastrointestinal bleeding, 2 due to liver failure, 2 due to multiple organ dysfunction syndrome, and 1 each due to HCC, sepsis, and hypophysoma; the cause was unknown in 5 patients.

### Cytokine Levels in Hepatic and Portal Veins

**Figure 1:**
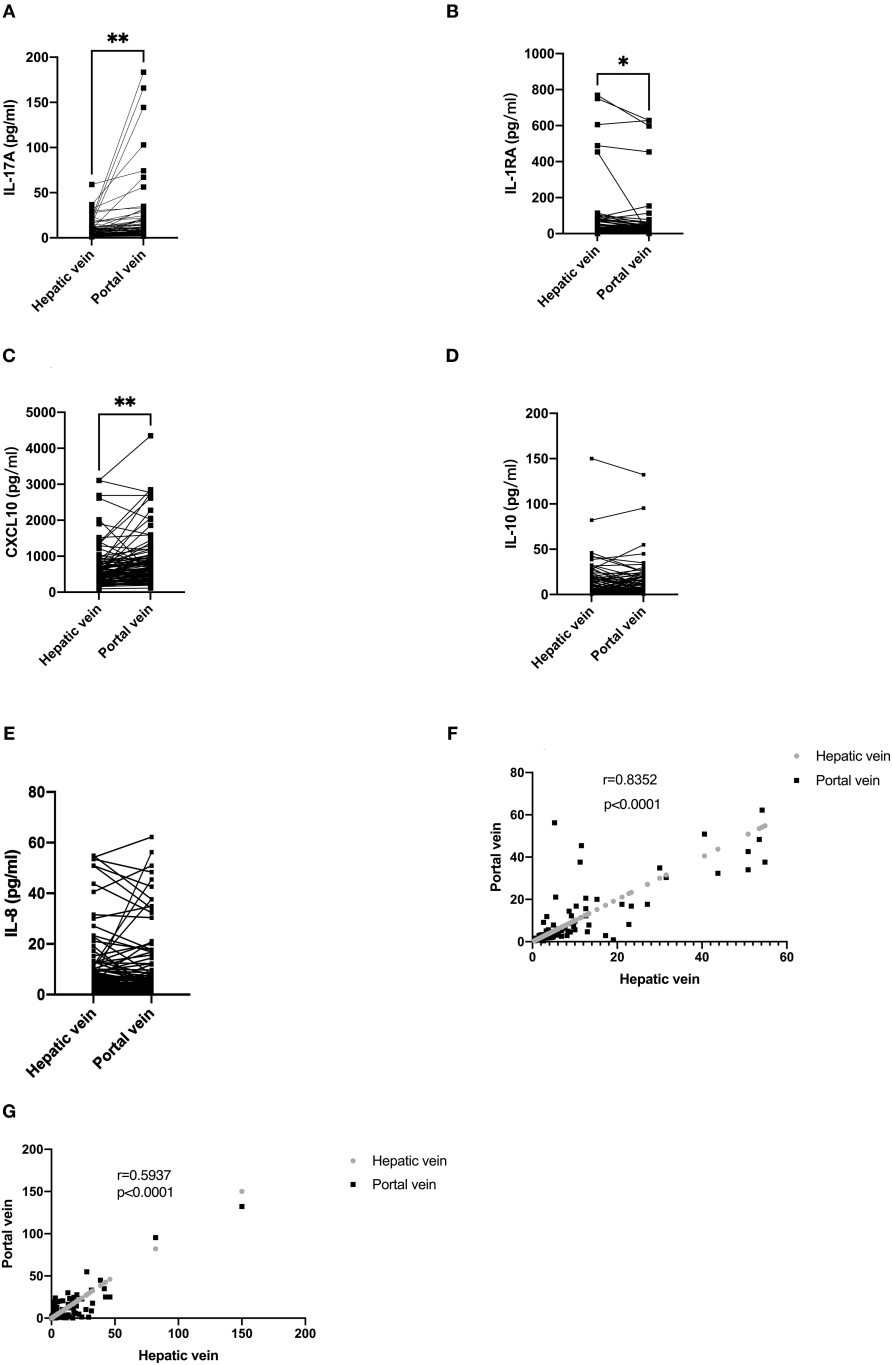
Serum levels of cytokines in the portal and hepatic veins and correlation between portal and hepatic veins. **(A)** The level of IL-17A in the portal vein was higher than in the hepatic vein. **(B)** The level of IL-1RA in the hepatic vein was higher than in the portal vein. **(C)** The level of CXCL10 in the portal was higher than in the hepatic vein. **(D,E)** There is no difference in IL-8 and IL-10 levels between the portal and hepatic veins. **(F,G)** IL-8 and IL-10 levels in portal and hepatic veins demonstrated a strong correlation. In **(A–E)**, data are shown paired with the Wilcoxon test used for comparison. In **(F,G)**, data are correlated via Spearman's correlation.

The level of cytokines in the hepatic and portal veins are displayed in Table 2. The levels of IL-17A [5.63 (3.17–12.77) vs. 5.20 (2.93–10.16) pg/ml; *P* < 0.01] and CXCL10 [645.01 (385.23–952.54) vs. 520.88 (354.97–759.61) pg/ml; *P* < 0.01] were higher in the portal than in the hepatic vein, respectively (Figures 1A,C). Conversely, IL-1RA displayed higher levels in the hepatic than in the portal veins [9.17 (1.22–28.54) vs. 11.56 (1.51–47.20 pg/ml, respectively; *P* < 0.05; Figure 1B). The differences in CXCL10 levels between the portal and hepatic veins was consistent with a previous study (10); however, no differences were found regarding IL-8 [4.27 (2.35–10.01) vs. 3.29 (1.96–9.45) pg/ml; *P* = 0.9658] or IL-10 [4.48 (1.96–14.43) vs. 3.62 (1.32–12.59) pg/ml; *P* = 0.1941] levels between the hepatic and portal veins (Figures 1D,E). Furthermore, the IL-10 (*r* = 0.5937, *P* < 0.0001) and IL-8 (*r* = 0.8352, *P* < 0.0001) levels at both sites were strongly correlated (Figures 1F,G).

**Table 2:** The level of cytokines in the hepatic vein and portal vein.

**Cytokines (pg/ml)**	**Hepatic vein**	**Portal vein**	***P-*value**
IL-10	4.48 (1.96–14.43)	3.62 (1.32–12.59)	0.194
IL-17A	5.20 (2.93–10.16)	5.63 (3.17–12.77)	0.006
IL-1RA	11.56 (1.51–47.20)	9.17 (1.22–28.54)	0.012
IL-8	4.27 (2.35–10.01)	3.29 (1.96–9.45)	0.966
CXCL10	520.88 (354.97–759.61)	645.01 (385.23–952.54)	0.001

*Data are shown as median and ranges. P-value is calculated by paired Wilcoxon test*.

### Prognostic Factors for Survival

Parameters affecting survival time were identified via univariate Cox regression analysis, and are depicted in Table 3. Higher IL-8 serum levels in hepatic venous blood before the TIPS procedure, and higher IL-10 serum levels in the portal vein, were significantly associated with poor survival in the univariate analysis. Child–Pugh scores and biochemical parameters—including the level of alanine aminotransferase and blood urea nitrogen—were also associated with poor survival. Multivariate analysis with parameters before the TIPS procedure presented Child–Pugh scores (*P* = 0.017, HR 1.484, 95% CI: 1.072–2.055) and the IL-8 level in the hepatic veins higher than 5.87 pg/ml (*P* < 0.001, HR 1.043, 95% CI: 1.019–1.068) as independent predictors (Table 3); however, no cytokines were associated with variceal rebleeding and hepatic encephalopathy in multivariate logistic regression analysis (Supplementary Tables 1, 2).

**Table 3:** Parameters correlating with survival in univariate Cox-regression analysis and in multivariate Cox-regression time-to-event analysis.

**Parameters**	**Univariate analysis**			**Multivariate analysis**		
	***P*-value**	**HR**	**Confidence interval**	***P*-value**	**HR**	**Confidence interval**
Age	0.188	1.027	0.987–1.068			
Child-pugh score	0.001	1.648	1.276–2.129	0.017	1.484	1.072–2.055
PPG before TIPS	0.087	1.090	0.987–1.202			
PVT	0.662	1.235	0.479–3.185			
Platelet count	0.218	0.992	0.980–1.005			
Serum TBil before TIPS	0.001	1.039	1.018–1.061			
Serum albumin before TIPS	0.004	0.894	0.827–0.965			
Serum ALT before TIPS	0.001	1.017	1.007–1.028	0.400	1.006	0.993–1.019
Serum Cr before TIPS	0.347	1.009	0.991–1.027			
Serum BUN before TIPS	0.002	1.228	1.076–1.400	0.277	1.095	0.930–1.289
INR before TIPS	0.003	24.902	3.076–201.610			
Hepatic levels of IL−10 before TIPS	0.066	1.012	0.999–1.026	0.072	1.013	0.999–1.028
Hepatic levels of IL-17A before TIPS	0.410	1.020	0.972–1.071			
Hepatic levels of IL-1RA before TIPS	0.919	1.000	0.997–1.003			
Hepatic levels of IL-8 before TIPS	0.001	1.040	1.018–1.063	0.0004	1.043	1.019–1.068
Hepatic levels of CXCL10 before TIPS	0.245	1.000	1.000–1.001			
Portal levels of IL-10 before TIPS	0.012	1.016	1.004–1.029			
Portal levels of IL-17A before TIPS	0.260	1.006	0.996–1.016			
Portal levels of IL-1RA before TIPS	0.568	1.001	0.998–1.004			
Portal levels of IL-8 before TIPS	0.001	1.038	1.016–1.061			
Portal levels of CXCL10 before TIPS	0.360	1.000	1.000–1.001			

*PPG, portal systemic pressure gradient; TBil, total bilirubin; ALT, alanine transaminase; INR, international normalized ratio; BUN, blood urea nitrogen; PVT, portal vein thrombosis; Cr, creatinine; TIPS, transjugular intrahepatic portosystemic shunt; HR, hazard ratio*.

### IL-8 Levels in Hepatic Veins Stratified by Etiology, Child–Pugh Scores, and Association With PPG

**Figure 2:**
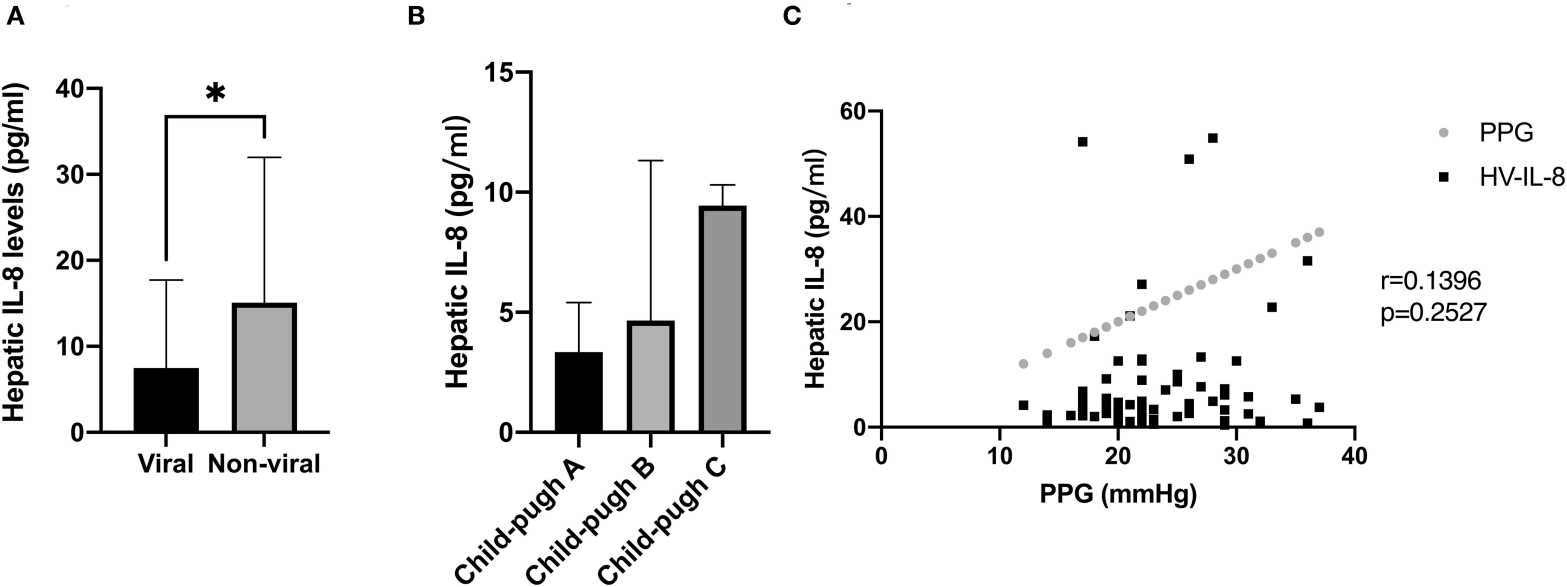
Association between IL-8 level and the severity of cirrhosis, etiology of cirrhosis, and portal hypertension. **(A)** Patients with viral induced cirrhosis displayed lower hepatic IL-8 levels than patients with non-viral cirrhosis. **(B)** Patients with class B and C cirrhosis demonstrated higher levels of IL-8 in the hepatic veins than patients with class A cirrhosis. **(C)** There was no correlation between hepatic IL-8 level and PPG before the TIPS procedure. In **(A,B)**, data were shown as mean and SEM, and the Mann-Whitney test and Kruskal-Wallis test were used for unpaired comparison. In **(C)**, data were correlated by Spearman's correlation analysis. *p < 0.05.

Hepatitis B virus (HBV) was the main cause of cirrhosis in our patients; hence, patients were stratified into viral and non-viral groups. Interestingly, patients with viral-induced cirrhosis displayed lower hepatic IL-8 levels than patients with non-viral cirrhosis (Figure 2A). When stratified by Child–Pugh classes, patients in class B and C showed higher IL-8 levels in hepatic veins than patients in class A (*P* = 0.065) (Figure 2B). There was no correlation with PPG regarding hepatic IL-8 levels before the TIPS procedure (Figure 2C).

### Association Between Hepatic IL-8 and Survival

**Figure 3:**
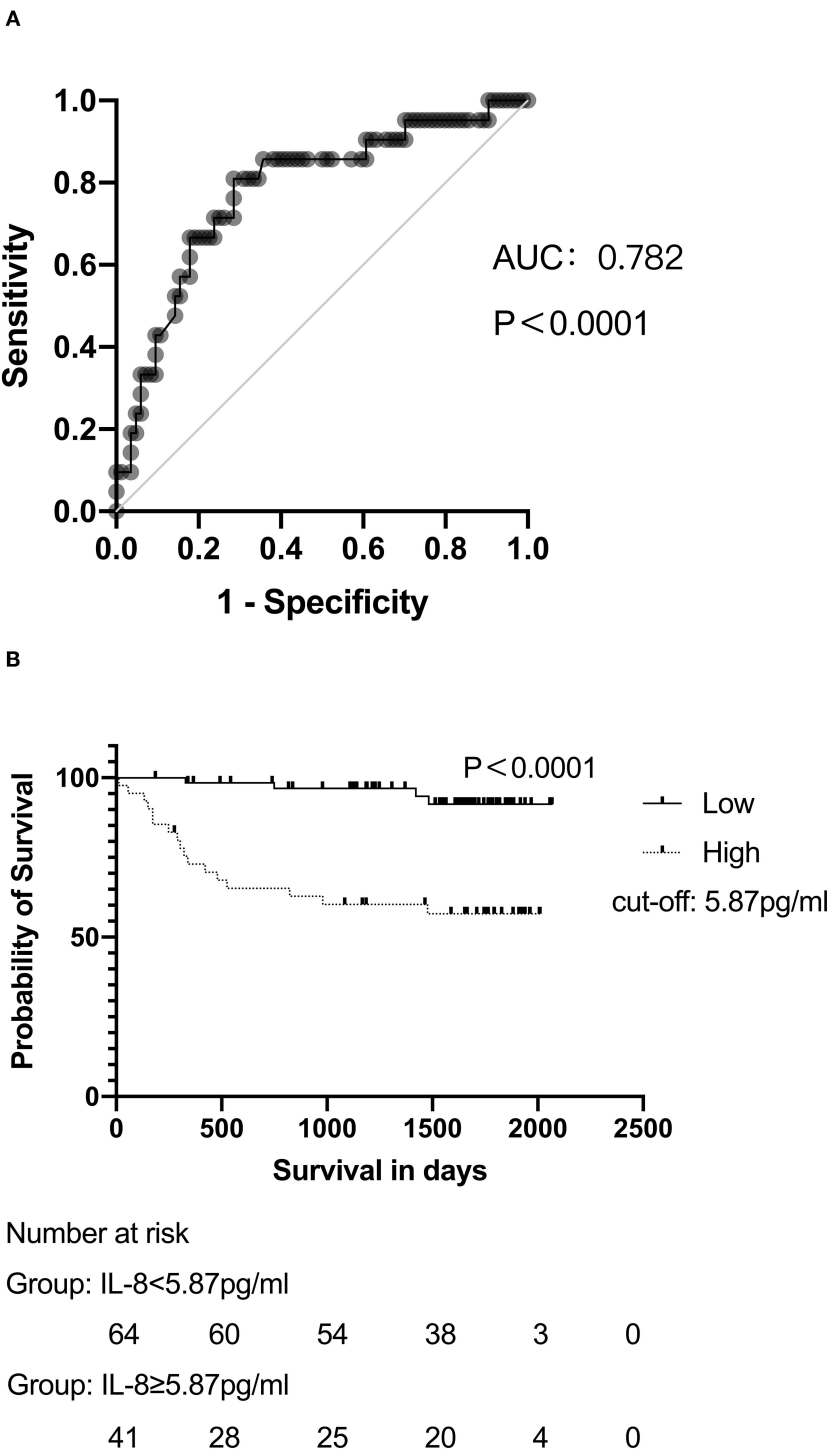
IL-8 levels are associated with the survival of patients with cirrhosis undergoing the TIPS procedure. **(A)** ROC analyses demonstrated the predictive value of hepatic IL-8 levels before the TIPS procedure. **(B)** Patients with hepatic IL-8 levels >5.87 pg/mL presented lower survival rates than patients with lower hepatic IL-8 levels after long-term follow up. In **(B)**, data were analyzed with the log-rank test and depicted as Kaplan–Meier curves.

Receiver operator characteristic analysis was performed to assess the predictive value of hepatic IL-8 levels for 4-year overall survival. IL-8 levels in the hepatic veins had a strong predictive value for survival in patients undergoing the TIPS procedure, with an area under the curve >0.78 (Figure 3A). The cut-off IL-8 level in the hepatic veins for survival was 5.87 pg/ml; patients with hepatic IL-8 levels <5.87 pg/ml had a significantly higher cumulative survival rate (98.4 vs. 72.9% at 1 year, 98.4 vs. 65.3% at 2 years, 96.7 vs. 60.3% at 3 years, 94.2 vs. 60.3% at 4 years; *P* < 0.0001; Figure 3B). Furthermore, higher hepatic IL-8 levels were associated with death in patients with child-Pugh A and B, while no significant difference was observed in child-Pugh C (Supplementary Figure 1).

## Discussion

Our study demonstrated the levels of five cytokines (IL-10, IL-17A, IL-1RA, IL-8, and CXCL10) from the portal and hepatic veins of patients undergoing the TIPS procedure. We identified that higher IL-8 levels in the hepatic vein were independently associated with mortality in patients undergoing the TIPS procedure. Recent investigations suggest that SI plays a major role in the pathogenesis of decompensation cirrhosis (4). Acute decompensation occurs in the setting of chronic SI due to translocation of proinflammatory molecules from the intestine or, damage-associated molecular patterns from the diseased liver or another organs (4). IL-6—a SI marker—was previously associated with the risk of first decompensation, and death or transplantation, in patients with compensated and decompensated advanced chronic liver disease, respectively (5). Similarly, it was found that the hepatic venous levels of soluble TNF-a receptors measured after the TIPS procedure may predict mortality in patients with severe portal hypertension (24).

Previous studies have demonstrated that CXCR3 ligands (CXCL9, CXCL10, and CXCL11) had significant predictive value for the long-term survival of patients receiving a TIPS (8–10). Our study enrolled 105 patients with severe portal hypertension who received a TIPS, evaluated five cytokines involved in SI in patients with cirrhosis, and demonstrated that the level of IL-8 in the hepatic vein had a strong predictive value for long-term survival in patients receiving TIPS.

IL-8, a proinflammatory CXC chemokine secreted by activated monocytes and macrophages in response to various stimuli (including inflammatory signals, and chemical and environmental stresses), acts on the CXCR1 and CXCR2 receptors (25, 26). Elevated IL-8 levels are associated with several diseases—including obesity, sepsis, atherosclerosis, and various forms of liver injury—suggesting that IL-8 may play a key role in the development and/or progression of these diseases by causing inflammation and tissue injury (15–18, 27). Previous studies demonstrated that circulating IL-8 levels in patients with cirrhosis were higher than in healthy controls, associated with the severity of cirrhosis, and involved in SI and the progress of chronic liver disease (19, 27). Furthermore, IL-8 was demonstrated to be an independent predictor for the presence of acute-on-chronic liver failure, and was strongly associated with 28- and 90-day mortality (12). However, few previous reports focus on IL-8 levels in the portal and hepatic veins of patients undergoing the TIPS procedure, or its predictive value for long-term survival.

Corresponding with previous data demonstrating that serum IL-8 levels increased with the pathogenesis of cirrhosis, our study revealed that patients with class B or C cirrhosis displayed higher hepatic IL-8 levels than those with class A (12). Consistent with the previous study, our study indicated that there was no correlation between IL-8 levels and hemodynamic parameters in patients with cirrhosis (27); this suggests that there was no cause-and-effect relationship between IL-8 and the hyperdynamic state when undergoing the TIPS procedure. This also suggested that the major factor associated with the rise in serum IL-8 levels in patients with cirrhosis was the progression of hepatic failure, and/or increased stimulation by TNF-a, rather than the presence of portal hypertension alone (20). Moreover, our study identified the predictive value of hepatic IL-8 levels for long-term survival in patients with cirrhosis receiving TIPS, which may be a result of the correlation between IL-8 levels and liver function.

Unlike CXCL10 (10), our results revealed that patients with viral-induced cirrhosis displayed lower hepatic IL-8 levels than patients with non-viral cirrhosis. Although the reason for this phenomenon was unknown, it may be partly related to the level of lipid accumulation in the liver in patients with non-viral cirrhosis. In patients with non-viral cirrhosis, most had non-alcoholic steatohepatitis or alcohol hepatitis in our cohort. Previous studies illustrated that lipid accumulation in hepatocytes induced the production of IL-8, which was a significant discriminator of non-alcoholic steatohepatitis severity due to its association with the advancement of steatosis (16, 21, 22), which may partly explain why the level of IL-8 in our cohort is lower than those from previous western studies. Interestingly, our study showed while there was no difference in the level of IL-8 between portal and hepatic veins, there was an excellent correlation between them; this might suggest that it was not the local inflammation of portal system and the IL-8 clearance decreased in patients with cirrhosis receiving TIPS. Notably, there was no overt infection among the included patients, a prerequisite for TIPS placement. Accordingly, the levels of IL-8 were much lower than in previous studies, suggesting that IL-8 was also an infection-independent indicator of survival.

Regarding CXCL10, our results differed from those of the previous study (10), which demonstrated that circulating CXCL10 could predict survival. This difference may partly result from the etiology, indication for the TIPS procedure, or ethnicity of the patients. Most patients had a cirrhotic etiology of HBV, while the main indication for a TIPS was variceal bleeding. We also revealed differences in IL-17A and IL-1RA levels between the portal and hepatic veins. IL-17A is a newly-discovered factor secreted by T-helper 17 lymphocytes (23), associated with the occurrence and development of various liver diseases (28, 29). Our study revealed that IL-17A levels were higher in the portal than hepatic veins of patients undergoing the TIPS procedure—consistent with CXCL10—suggesting that circulating IL-17A did not mainly derive from hepatic immune cells, but rather from immune cells in the splanchnic circulation; conversely, IL-1RA levels were higher in the hepatic than portal veins, implying that IL-1RA may be synthesized by liver immune cells.

Our study has several limitations; first, it is limited by the retrospective, single-center design, as well as the lack of a control group. However, it would be particularly difficult to acquire blood samples from the portal and hepatic veins of healthy individuals.

Second, the level of cytokines in the hepatic or portal veins following the TIPS procedure was not measured. The small number of cytokines is another limitation in our study, which may not accurately reflect the state of SI in patients with cirrhosis.

In conclusion, our study demonstrates that lower IL-8 levels in the hepatic veins were significantly associated with higher cumulative survival rates in patients with cirrhosis undergoing the TIPS procedure. IL-8—a biomarker of SI—is widely involved in the pathogenesis of decompensated liver disease. These results suggest an association between hepatic inflammation and clinical outcomes in patients with cirrhosis treated with TIPS. Further research revealing the role of IL-8 in patients with advanced liver disease may thus improve treatment, allowing for more precise and personalized care in these patients.

## Data Availability Statement

The raw data supporting the conclusions of this article will be made available by the authors, without undue reservation.

## Ethics Statement

The studies involving human participants were reviewed and approved by the Ethical Review Board of West China Hospital. The patients/participants provided their written informed consent to participate in this study and for the publication of any potentially identifiable images or data included in this article. Patients also provided written informed consent for the transjugular intrahepatic portosystemic shunt procedures.

## Author Contributions

XL conceived and designed the study. GL, XW, and TY performed the research. YY and TX contributed the data acquisition. GL and XW conducted data analysis/interpretation and wrote the manuscript. TY contributed statistical analysis. LY provided clinical advice. All authors have read and approved the final manuscript.

## Funding

The project is supported by 1·3·5 project for disciplines of excellence–Clinical Research Incubation Project, West China Hospital, Sichuan University (2019HXFH055); Project for Science and Technology Department of Sichuan Province (2020YFH0089); The China Postdoctoral Science Foundation (2020M683326).

## Conflict of Interest

The authors declare that the research was conducted in the absence of any commercial or financial relationships that could be construed as a potential conflict of interest.

## Publisher's Note

All claims expressed in this article are solely those of the authors and do not necessarily represent those of their affiliated organizations, or those of the publisher, the editors and the reviewers. Any product that may be evaluated in this article, or claim that may be made by its manufacturer, is not guaranteed or endorsed by the publisher.

## Supplementary Material

Supplementary Figure 1 The relationship between IL-8 levels and survival in patients with Child-Pugh **(A)**, Child-Pugh **(B)**, and Child-Pugh **(C)**. Higher hepatic IL-8 levels were associated with death in patients with Child-Pugh **(A)**. Higher hepatic IL-8 levels were associated with death in patients with Child-Pugh **(B)**. No difference in hepatic IL-8 levels between patients with alive and dead was observed in Child-Pugh **(C)**.

Supplementary Table 1 Parameters correlating with hepatic encephalopathy in univariate logistic regression analysis and in multivariate logistic regression analysis.

Supplementary Table 2 Parameters correlating with variceal rebleeding in univariate logistic regression analysis and in multivariate logistic regression analysis.

## Abbreviations

TIPS: transjugular intrahepatic portosystemic shunt
SI: systemic inflammation
IL: interleukin
PPG: portosystemic pressure gradient
HBV: hepatitis B virus.
