# Supplementary material for: High Interleukin-8 Levels Associated With Decreased Survival in Patients With Cirrhosis Following Transjugular Intrahepatic Portosystemic Shunt
Source: Front Med (Lausanne). 2022 Feb 28;9:829245. doi: 10.3389/fmed.2022.829245 (PMC8918632; doi:10.3389/fmed.2022.829245)
Supplement: Supplementary Table 1 — Parameters correlating with hepatic encephalopathy in univariate logistic regression analysis and in multivariate logistic regression analysis. [file Table_1.DOCX]

**Supplementary Table 1.** parameters correlating with hepatic encephalopathy in univariate logistic regression analysis and in multivariate logistic regression analysis.

| Parameters | univariate logistic regression analysis | | | multivariate logistic regression analysis | | |
| --- | --- | --- | --- | --- | --- | --- |
|  | P-value | OR | Confidence interval | P-value | OR | Confidence interval |
| Gender | 0.390 | 0.672 | 0.271-1.664 |  |  |  |
| Age | 0.026 | 1.045 | 1.005-1.087 | 0.044 | 1.043 | 1.001-1.087 |
| Child-pugh score | 0.947 | 0.991 | 0.766-1.283 |  |  |  |
| Infection after TIPS | 0.706 | 0.824 | 0.302-2.247 |  |  |  |
| Ascites before TIPS | 0.488 | 0.727 | 0.296-1.790 |  |  |  |
| PPG before TIPS | 0.131 | 0.931 | 0.848-1.022 |  |  |  |
| Serum TBil before TIPS | 0.718 | 0.994 | 0.963-1.027 |  |  |  |
| Serum albumin before TIPS | 0.941 | 1.003 | 0.935-1.076 |  |  |  |
| Serum ALT before TIPS | 0.925 | 1.001 | 0.985-1.016 |  |  |  |
| Serum Cr before TIPS | 0.071 | 1.021 | 0.998-1.045 | 0.185 | 1.015 | 0.993-1.039 |
| Serum BUN before TIPS | 0.550 | 1.046 | 0.902-1.213 |  |  |  |
| INR before TIPS | 0.128 | 0.144 | 0.012-1.743 |  |  |  |
| Hepatic levels of IL10 before TIPS | 0.153 | 1.019 | 0.993-1.045 |  |  |  |
| Hepatic levels of IL-17A before TIPS | 0.645 | 0.988 | 0.939-1.040 |  |  |  |
| Hepatic levels of IL-1RA before TIPS | 0.958 | 1.000 | 0.997-1.003 |  |  |  |
| Hepatic levels of IL8 before TIPS | 0.640 | 0.992 | 0.959-1.026 |  |  |  |
| Hepatic levels of CXCL10 before TIPS | 0.536 | 1.000 | 0.999-1.001 |  |  |  |
| Portal levels of IL10 before TIPS | 0.095 | 1.024 | 0.996-1.052 | 0.096 | 1.025 | 0.996-1.054 |
| Portal levels of IL-17A before TIPS | 0.491 | 0.995 | 0.979-1.010 |  |  |  |
| Portal levels of IL-1RA before TIPS | 0.600 | 1.001 | 0.997-1.004 |  |  |  |
| Portal levels of IL8 before TIPS | 0.588 | 0.991 | 0.961-1.023 |  |  |  |
| Portal levels of CXCL10 before TIPS | 0.574 | 1.000 | 1.000-1.001 |  |  |  |

PPG portal systemic pressure gradient, TBil total bilirubin, ALT alanine transaminase, INR international normalized ratio, BUN blood urea nitrogen, PVT portal vein thrombosis, Cr creatinine, TIPS transjugular intrahepatic portosystemic shunt, OR odds ratio.

**Supplementary Table 2.** parameters correlating with variceal rebleeding in univariate logistic regression analysis and in multivariate logistic regression analysis.

| Parameters | univariate logistic regression analysis | | | multivariate logistic regression analysis | | |
| --- | --- | --- | --- | --- | --- | --- |
|  | P-value | OR | Confidence interval | P-value | OR | Confidence interval |
| Gender | 0.685 | 0.822 | 0.319-2.118 |  |  |  |
| Age | 0.178 | 1.028 | 0.988-1.069 |  |  |  |
| Child-pugh score | 0.051 | 1.325 | 0.999-1.758 | 0.125 | 1.275 | 0.935-1.738 |
| Infection after TIPS | 0.497 | 0.682 | 0.227-2.054 |  |  |  |
| Ascites before TIPS | 0.399 | 1.556 | 0.557-4.343 |  |  |  |
| PPG before TIPS | 0.575 | 1.026 | 0.938-1.123 |  |  |  |
| Serum TBil before TIPS | 0.122 | 1.026 | 0.993-1.060 |  |  |  |
| Serum albumin before TIPS | 0.092 | 0.935 | 0.865-1.011 |  |  |  |
| Serum ALT before TIPS | 0.118 | 1.013 | 0.997-1.029 | 0.343 | 1.009 | 0.991-1.028 |
| Serum Cr before TIPS | 0.176 | 1.014 | 0.994-1.034 | 0.217 | 1.015 | 0.991-1.040 |
| Serum BUN before TIPS | 0.162 | 1.115 | 0.957-1.300 | 0.986 | 1.002 | 0.827-1.214 |
| INR before TIPS | 0.199 | 4.574 | 0.449-46.581 |  |  |  |
| Hepatic levels of IL10 before TIPS | 0.776 | 1.003 | 0.982-1.025 |  |  |  |
| Hepatic levels of IL-17A before TIPS | 0.714 | 0.990 | 0.938-1.045 |  |  |  |
| Hepatic levels of IL-1RA before TIPS | 0.342 | 0.998 | 0.993-1.003 |  |  |  |
| Hepatic levels of IL8 before TIPS | 0.684 | 0.993 | 0.958-1.029 |  |  |  |
| Hepatic levels of CXCL10 before TIPS | 0.713 | 1.000 | 0.999-1.001 |  |  |  |
| Portal levels of IL10 before TIPS | 0.473 | 1.008 | 0.986-1.031 |  |  |  |
| Portal levels of IL-17A before TIPS | 0.354 | 1.006 | 0.993-1.019 |  |  |  |
| Portal levels of IL-1RA before TIPS | 0.482 | 0.998 | 0.993-1.003 |  |  |  |
| Portal levels of IL8 before TIPS | 0.576 | 1.009 | 0.979-1.040 |  |  |  |
| Portal levels of CXCL10 before TIPS | 0.589 | 1.000 | 0.999-1.000 |  |  |  |

PPG portal systemic pressure gradient, TBil total bilirubin, ALT alanine transaminase, INR international normalized ratio, BUN blood urea nitrogen, PVT portal vein thrombosis, Cr creatinine, TIPS transjugular intrahepatic portosystemic shunt, OR odds ratio.
